# The frontal skull Hounsfield unit value can predict ventricular enlargement in patients with subarachnoid haemorrhage

**DOI:** 10.1038/s41598-018-28471-1

**Published:** 2018-07-05

**Authors:** Yu Deok Won, Min Kyun Na, Choong Hyun Kim, Jae Min Kim, Jin Hwan Cheong, Je Il Ryu, Myung-Hoon Han

**Affiliations:** Department of Neurosurgery, Hanyang University Guri Hospital, 153 Gyeongchun-ro, Guri, Gyonggi-do Korea

## Abstract

Hydrocephalus is a common complication following subarachnoid haemorrhage (SAH) arising from spontaneous aneurysm rupture. The Hounsfield unit (HU) value from computed tomography scans may reflect bone mineral density, which correlates with body mass index, which in turn is related to post-SAH ventricle size changes. We herein investigated potential associations between frontal skull HU values and ventricle size changes after SAH. HU values from four different areas in the frontal bone were averaged to minimize measurement errors. The bicaudate index and Evans ratio were measured using both baseline and follow-up CT images. CT images with bicaudate index >0.2 and Evans ratio >0.3 simultaneously were defined as indicating ventriculomegaly. We included 232 consecutive patients with SAH due to primary spontaneous aneurysm rupture, who underwent clipping over almost a 9-year period at a single institution. The first tertile of frontal skull HU values in older patients (≥55 years) was an independent predictor of ventriculomegaly after SAH, as compared to the third tertile in younger patients (hazard ratio, 4.01; 95% confidence interval 1.21–13.30; p = 0.023). The lower frontal skull HU value independently predicted ventricular enlargement post-SAH, due to the potential weak integrity of subarachnoid trabecular structures in younger patients.

## Introduction

Hydrocephalus is a common complication of subarachnoid haemorrhage (SAH) due to spontaneous aneurysmal rupture. The incidence of hydrocephalus after SAH reportedly ranges from approximately 20–30%^1^.

Body mass index (BMI) is thought to correlate with bone mineral density (BMD). In a recent evaluation of whether BMD is directly associated with ventricular enlargement after SAH, we showed that higher BMI may be an independent factor in the suppression of ventricle growth after SAH due to aneurysm rupture^2^. However, due to the retrospective nature of our study, T-score data of BMD were not available in the SAH patients, as BMD is usually not evaluated in SAH patients. On the other hand, previous studies have indicated that Hounsfield unit (HU) values obtained from computed tomography (CT) scans may be an alternative method for determining regional BMD^3–6^. Therefore, in the present study, we measured HU values in the frontal skull to evaluate the potential association with ventricular enlargement during the clinical course of SAH. Additionally, we sought other potentially predictive factors of ventriculomegaly. The Evans ratio and the bicaudate index (BCI), which are widely used in the neurosurgical field, were used to define ventricular enlargement in consecutive patients with aneurysmal SAH, who underwent clipping at a single institution.

## Results

### Patient characteristics

Two-hundred-and-thirty-two consecutive patients (>18 years old) with SAH due to primary spontaneous aneurysm rupture, who underwent clipping at a single institution over an almost 9-year period. There were 143 (61.1%) female patients, with a mean age of 54.8 years. The median frontal skull HU value was 843.3 in the younger age group (<55 years) and 707.8 in the older age group. Seventy-one (30.6%) patients were categorized as having ventricular enlargement from the follow-up CT images, based on the BCI and the Evans ratio. Further descriptive data are shown in Tables 1 and 2.

**Table 1:** Radiological characteristics of patients with spontaneous subarachnoid haemorrhage who underwent surgical treatment, grouped by age.

Characteristics	Age < 55 years (N = 127)	Age ≥ 55 years (N = 105)	Total (N = 232)	p
Skull HU value, median (IQR), HU	843.3 (685.0–1022.3)	707.8 (568.1–828.5)	773.0 (625.7–944.6)	<0.001
Classification of the skull HU value, based on tertile groups, HU				<0.001
Tertile 1	≤737.3	≤624.8	≤684.0	
Tertile 2	737.4–966.3	624.9–772.0	684.1–868.8	
Tertile 3	>966.3	>772.0	>868.8	
Evans ratio on admission, mean ± SD	0.253 ± 0.041	0.272 ± 0.036	0.262 ± 0.040	<0.001
BCI on admission, mean ± SD	0.127 ± 0.037	0.162 ± 0.040	0.143 ± 0.042	<0.001
Ventriculomegaly on admission, n (%)				
BCI > 0.2	5 (3.9)	17 (16.2)	22 (9.5)	0.001
Evans ratio > 0.3	13 (10.2)	21 (20.0)	34 (14.7)	0.028
BCI > 0.2 and Evans ratio > 0.3	4 (3.1)	10 (9.5)	14 (6.0)	0.040
Follow-up Evans ratio, mean ± SD	0.281 ± 0.061	0.299 ± 0.058	0.289 ± 0.060	0.022
Follow-up BCI, mean ± SD	0.166 ± 0.062	0.187 ± 0.059	0.176 ± 0.062	0.008
Follow-up ventriculomegaly, n (%)				
BCI > 0.2	41 (32.3)	47 (44.8)	88 (37.9)	0.035
Evans ratio > 0.3	33 (26.0)	43 (41.0)	75 (32.3)	0.017
BCI > 0.2 and Evans ratio > 0.3	32 (25.2)	39 (37.1)	71 (30.6)	0.034
Time duration between SAH and follow-up image, mean ± SD, days	109.5 ± 111.4	82.4 ± 95.2	97.2 ± 105.0	0.050

BCI, Bicaudate index; SAH, subarachnoid haemorrhage; SD, standard deviation.

**Table 2:** Clinical characteristics of patients with spontaneous subarachnoid haemorrhage who underwent surgical treatment, grouped by age.

Characteristics	Age < 55 years (N = 127)	Age ≥ 55 years (N = 105)	Total (N = 232)	p
Sex				<0.001
Female, n (%)	60 (47.2)	83 (79.0)	143 (61.6)	
Age, mean ± SD, years	45.8 ± 6.1	65.7 ± 7.9	54.8 ± 12.1	<0.001
Height, mean ± SD, cm	164.9 ± 8.3	157.4 ± 7.7	161.5 ± 8.9	<0.001
Weight, mean ± SD, kg	66.5 ± 12.5	58.2 ± 9.2	62.7 ± 11.9	<0.001
BMI, mean ± SD, kg/m^2^	24.3 ± 3.4	23.4 ± 3.1	23.9 ± 3.3	0.041
Hunt-Hess grade, n (%)				0.066
Grade 1	8 (6.3)	3 (2.9)	11 (4.7)	
Grade 2	62 (48.8)	43 (41.0)	105 (45.3)	
Grade 3	29 (22.8)	42 (40.0)	71 (30.6)	
Grade 4	26 (20.5)	16 (15.2)	42 (18.1)	
Grade 5	2 (1.6)	1 (1.0)	3 (1.3)	
Modified Fisher grade, n (%)				0.024
1	36 (28.3)	13 (12.4)	49 (21.1)	
2	21 (16.5)	21 (20.0)	42 (18.1)	
3	25 (19.7)	21 (20.0)	46 (19.8)	
4	45 (35.4)	50 (47.6)	95 (40.9)	
Location, n (%)				0.167
ACA	49 (38.6)	29 (27.6)	78 (33.6)	
MCA	43 (33.9)	32 (30.5)	75 (32.3)	
ICA	10 (7.9)	9 (8.6)	19 (8.2)	
PCOM	21 (16.5)	30 (28.6)	51 (22.0)	
VBA	4 (3.1)	5 (4.8)	9 (3.9)	
IVH, n (%)				0.054
No	61 (48.0)	34 (32.4)	95 (40.9)	
Focal	39 (30.7)	42 (40.0)	81 (34.9)	
Pan	27 (21.3)	29 (27.6)	56 (24.1)	
ICH, n (%)	32 (25.2)	31 (29.5)	63 (27.2)	0.556
Operation type, n (%)				0.448
Craniotomy	107 (84.3)	93 (88.6)	200 (86.2)	
Craniectomy	20 (15.7)	12 (11.4)	32 (13.8)	
Fenestration of the lamina terminalis, n (%)	52 (40.9)	35 (33.3)	87 (37.5)	0.291
Vasospasm, n (%)	26 (20.5)	21 (20.0)	47 (20.3)	1.000
Ventriculo-peritoneal shunt, n (%)	22 (17.3)	29 (27.6)	51 (22.0)	0.084

SD, standard deviation; BMI, body mass index; ACA, anterior cerebral artery; MCA, middle cerebral artery; ICA, internal carotid artery; PCOM, posterior communicating artery; VBA, vertebrobasilar artery; IVH, intraventricular haemorrhage; ICH, intracerebral haemorrhage.

### Association between skull HU and age

**Figure 1:**
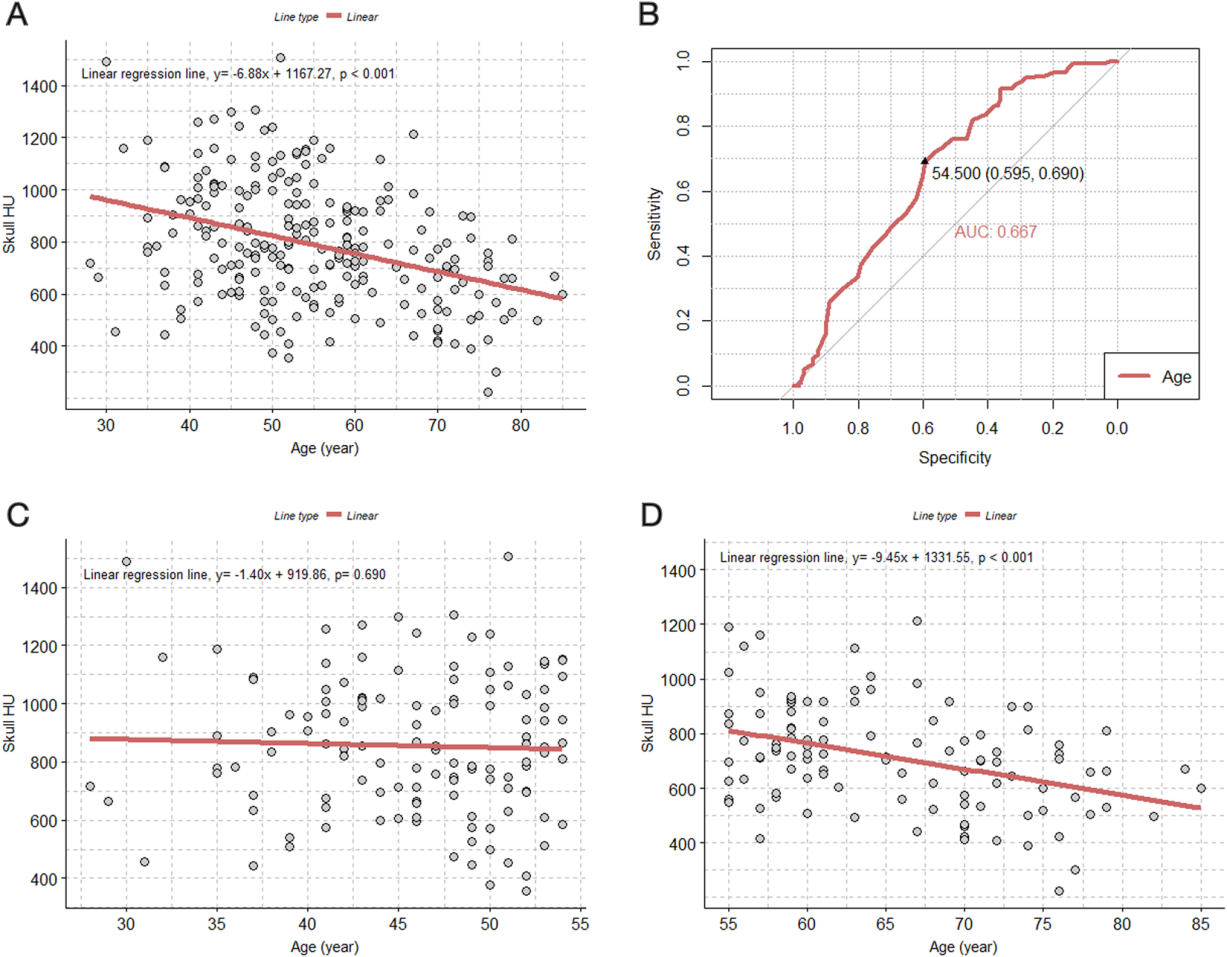
Scatterplot with a linear regression line and receiver operating characteristic curve for evaluating the association between age and skull HU values. (**A**) Association between age and skull HU values in all patients; (**B**) receiver operating characteristic curve for the dependent variable of the skull HU value, divided by the median to determine the optimal cut-off age value; (**C**) association between age and skull HU value in the younger age group (under 55 years old); (**D**) association between age and skull HU values in patients who are 55-years-old or older. HU = Hounsfield unit.

There was an overall significant negative association between the frontal skull HU value and age (Fig. 1A). Figure 1A shows that the HU value decreased by approximately 7 HU per 1-year increase in age (β = −6.88; p < 0.001). According to the receiver operating characteristic (ROC) curve, the optimal cut-off value for age was 54.5 years for the prediction of lower median skull HU values (<773.0) (Fig. 1B). When we divided patients into two age groups (<55, and ≥55 years) according to the ROC curve, the association between the skull HU value and age was only significant in the older age group (β = −9.45; p < 0.001). The associations between each of the four HU values of the frontal bone and age are shown in Supplementary Fig. S1.

### Trend in ventricle size change after SAH

**Figure 2:**
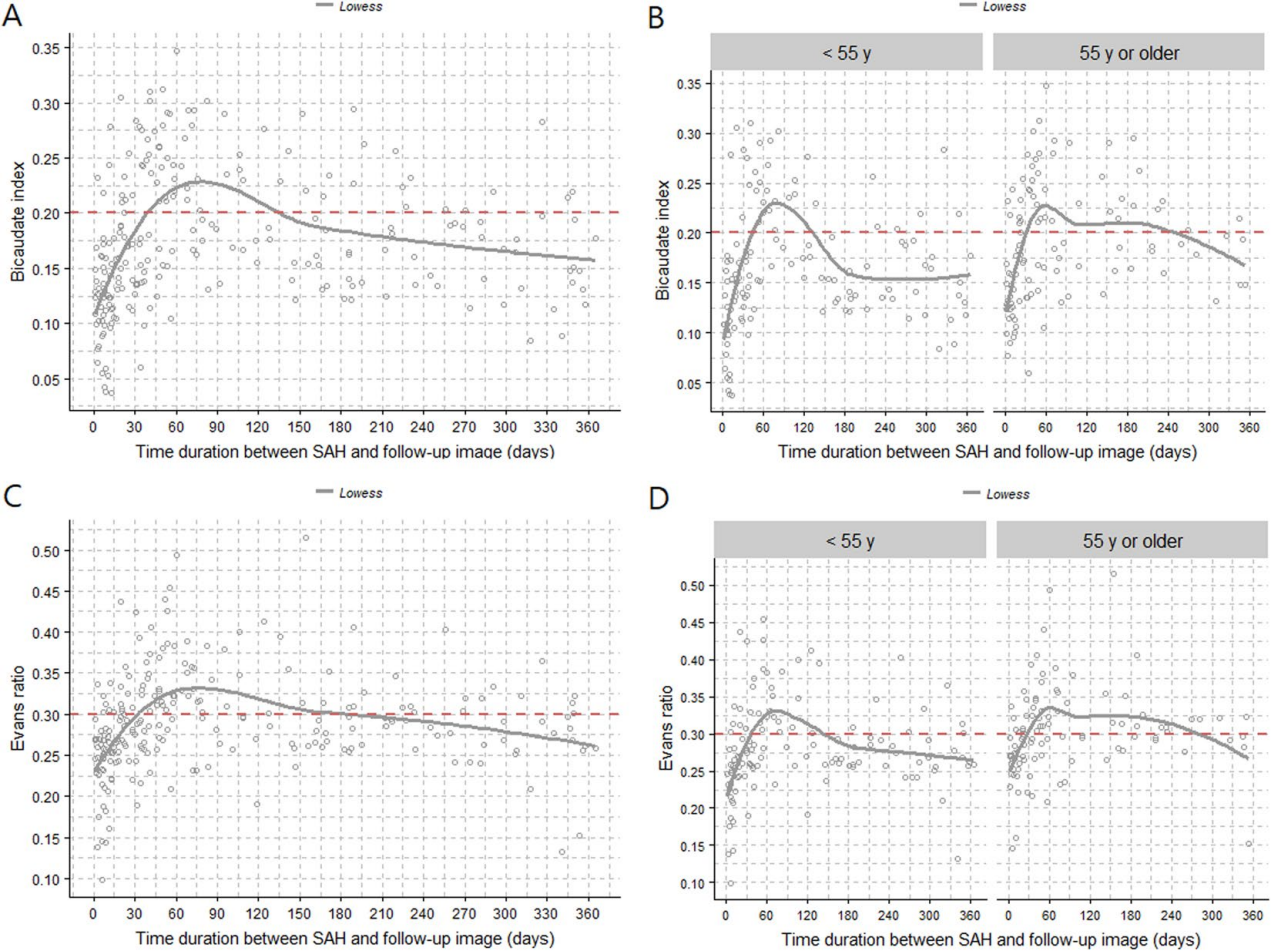
Scatterplot with LOWESS curve showing the trend between time after SAH and the BCI and Evans ratio. (**A**) BCI and time after SAH in all patients. (**B**) BCI and time after SAH based on the age group (<55 years and ≥55 years). (**C**) Evans ratio and time after SAH in all patients. (**D**) Evans ratio and time after SAH based on the age group (<55 years and ≥55 years). BCI = bicaudate index; SAH = subarachnoid haemorrhage.

Although it was not possible to present the precise natural course of ventricle size changes after SAH due to the irregular time intervals between the occurrence of SAH and the follow-up CT (22% of the patients also received a ventriculo-peritoneal [V-P] shunt), we sought to evaluate the overall changes in ventricle size after SAH. We observed an overall rapid ventricular enlargement until around 2 months after SAH (Fig. 2A and C). The overall ventricle size then decreased gradually, until at 4–5 months after SAH, it was below the ventriculomegaly threshold based on the BCI and the Evans ratio. When we divided the patients into two age groups, we found a relatively abrupt decrease in ventricle size by 2 or 3 months after SAH in the younger patient group. However, ventricular dilatation continued until around 8–9 months after SAH in the older patient group (Fig. 2B and D).

### Association of the skull HU value and ventriculomegaly after SAH

**Figure 3:**
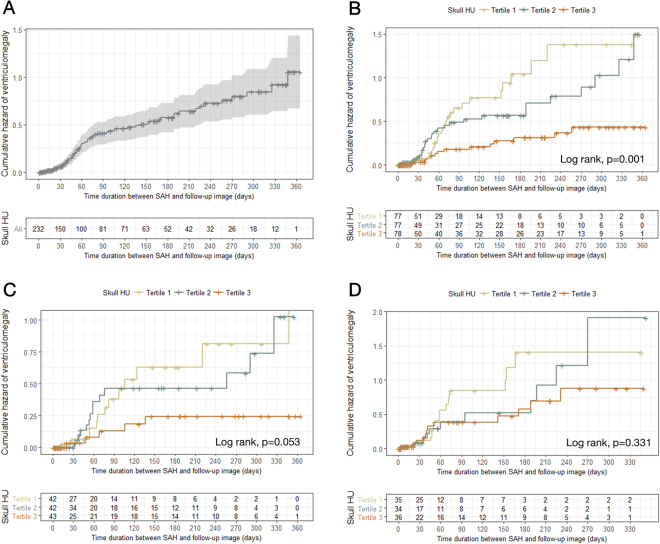
Kaplan–Meier curves showing the cumulative risk for ventriculomegaly. (**A**) Overall patients; (**B**) overall patients based on tertile groups of skull HU values; (**C**) younger age group based on tertile groups of skull HU values; (**D**) older age group based on tertile groups of skull HU values. HU = Hounsfield unit.

We present the direct relationship between skull bone density and follow-up ventricle size after SAH, based on age, in Supplementary Fig. S2A–C. Figure 3A shows the overall development of ventriculomegaly within 1 year after SAH. We observed that the patients falling in the first (≤684.0) and second tertile (684.1–868.8) of the frontal skull HU values showed significantly greater ventriculomegaly development than those in the third tertile (>868.8) (Fig. 3B). In the younger patient group (<55 years), the first (≤737.3) and second tertile (737.4–966.3) of frontal skull HU value groups also showed a tendency for increased ventriculomegaly development, although this did not quite reach statistical significance (p = 0.053) (Fig. 3C). However, there was no significant association between ventriculomegaly and skull HU value in the older age group (p = 0.331) (Fig. 3D).

In the multivariate Cox regression analysis of all patient data, individuals within the second tertile of skull HU values showed a 2.4-fold higher risk of ventriculomegaly than those in the third tertile group (HR, 2.40; 95% CI 1.22–4.75; p = 0.011) (Supplementary Fig. S3). The first tertile group of skull HU values demonstrated ca. 1.9-fold increased risk of ventriculomegaly, although this increase did not reach statistical significance (p = 0.085).

**Figure 4:**
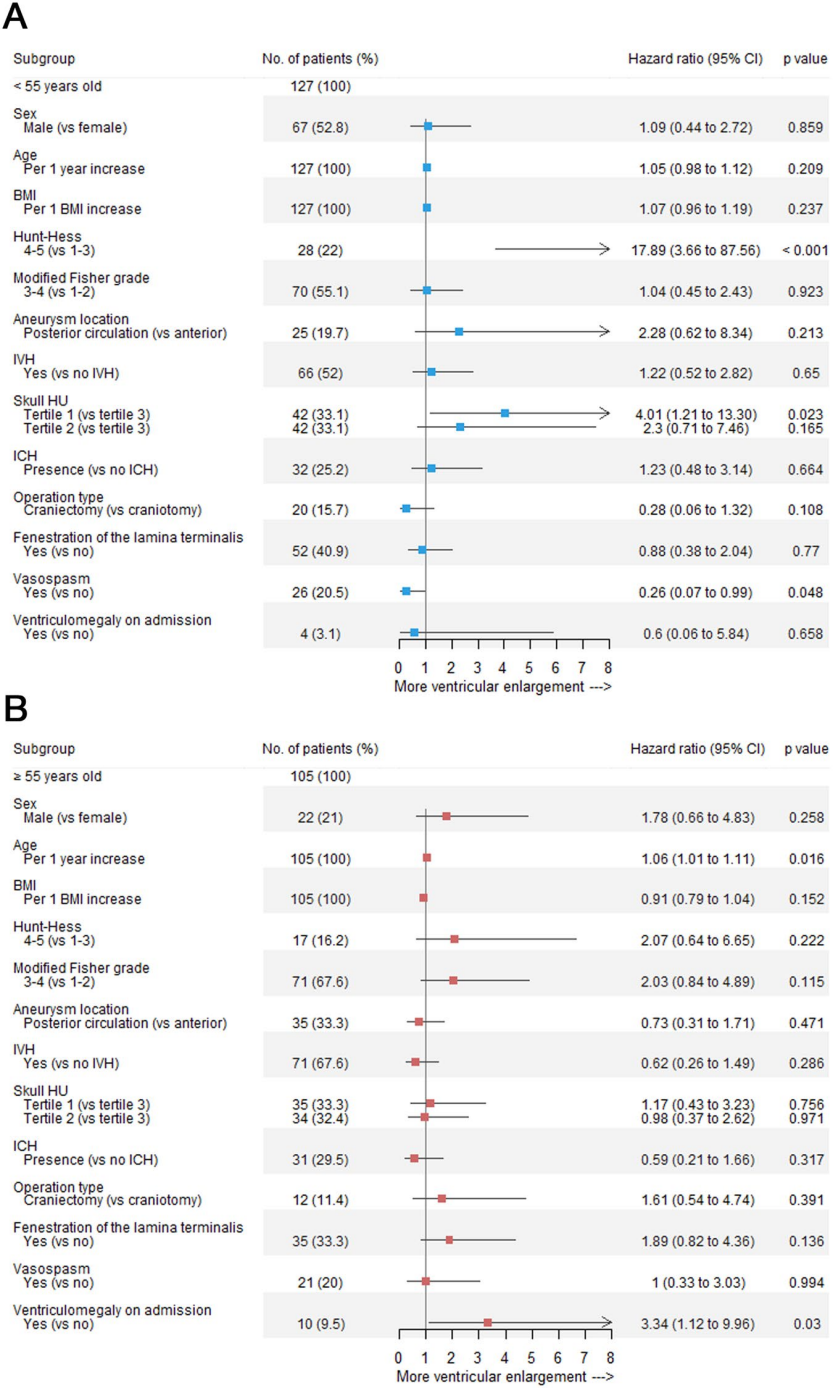
Forest plots of estimates from the multivariate Cox regression of ventriculomegaly according to the potential predictive factors (adjusted for sex, age [continuous variable], BMI [continuous variable], Hunt–Hess grade, modified Fisher grade, aneurysm location, IVH, skull HU values, ICH, operation type, fenestration of the lamina terminalis, vasospasm, and ventriculomegaly on admission). (**A**) Younger age group; (**B**) older age group. BMI = body mass index; IVH = intraventricular haemorrhage; HU = Hounsfield unit; ICH = intracerebral haemorrhage.

However, the association between age and skull HU values may have affected these results. When we performed the analysis again according to age group classifications, the first tertile skull HU values independently predicted ventricular enlargement after SAH, as compared to the third tertile, in the younger individuals (HR, 4.01; 95% CI 1.21–13.30; p = 0.023) (Fig. 4A). There was no significant association between skull HU values and ventriculomegaly in the older age group (Fig. 4B). Further univariate Cox regression analysis results are shown in Supplementary Tables S1–3

### Other factors associated with ventricular enlargement

The initial Hunt–Hess grade showed a strong correlation with ventriculomegaly development (HR, 17.89; 95% CI 3.66–87.56; p < 0.001) in the younger age group after full adjustment for predictive variables (Fig. 4A). Age was associated with ventriculomegaly in the older age group. There was about a 1.06-fold increase in ventriculomegaly per 1-year increase in age (HR, 1.06; 95% CI 1.01–1.11; p = 0.016). In addition, ventriculomegaly on admission was associated with continuous ventricular dilatation at follow-up in the older age group (Fig. 4B).

## Discussion

Our study showed the association between ventriculomegaly with/without hydrocephalus-related symptoms after SAH and the HU values of the cancellous bone in the frontal skull in individuals under 55 years old. The group comprising the first tertile of skull HU values showed an approximate 4-fold increased risk of ventricular enlargement after SAH as compared to the third tertile group in those less than 55 years old, after adjusting for other predictive factors.

The HU value is obtained from a linear transformation of the measured attenuation coefficients, according to the following equation: Hounsfield units, HU = [1000 × (µ_voxel_ − µ_water_)]/µ_water_ (µ_voxel_ is the calculated voxel attenuation coefficient and µ_water_ is the attenuation coefficient of water). Any anatomic region on a CT image has an accurate and absolute HU value, which demonstrates validity and reproducibility in all appropriately calibrated CT scanners^7,8^. Previous studies have reported that the HU value of the trabecular (cancellous bone) area of the lumbar spine decreases with age and is positively correlated with BMD and the T-score^3,4,9^. A flat bone of the cranium has inner and outer layers made of cortical bone; in between these layers is a region called the diploe, which is made up of cancellous bone^10^. Therefore, we hypothesized that the HU value of the trabecular area of the skull would also be associated with age and systemic BMD.

The space between the arachnoid and the pia mater is filled with CSF and contains abundant strands of collagen tissue that connects the arachnoid to the pia mater; these are known as trabeculae. Arachnoid granulations allow CSF clearance from the subarachnoid space to the venous sinus^11^. A previous human *ex vivo* study also confirmed that CSF outflow occurs across human arachnoid granulations^12^. The core of an arachnoid granulation is composed of arachnoid trabeculae^13^, which are composed of type 1 collagen. Other type 1 collagen tissues are skin, tendon, vascular ligatures, organs, and bone.

Osteoporosis is a systemic disease that affects the systemic bone mass and microarchitecture throughout the body^14^. Moreover, osteoporosis is strongly associated with *COL1A1* and *COL1A2*, genes that encode type 1 collagen components^15^. Osteogenesis imperfecta, known as a collagen type 1 disease, is associated with communicating hydrocephalus, intracranial aneurysm, and arterial dissection^16–18^. Therefore, we hypothesized that lower BMD, which is indicated by the HU value of the cancellous bone of the frontal skull, and which probably has a strong genetic underpinning, may negatively influence the integrity of arachnoid trabeculae, which are composed of type 1 collagen. When SAH occurs, the acute haemorrhage in the subarachnoid space damages the arachnoid granulations and trabeculae. After the haematoma resolves, the integrity of the arachnoid granulations and trabeculae may be relatively less damaged in individuals with dense and strong type 1 collagen tissue, and this may be predicted by systemic BMD, which is also related to type 1 collagen. If the arachnoid granulations have fairly good integrity, efficient CSF outflow to the venous sinus can be maintained, reducing the likelihood that communicating hydrocephalus will develop, as compared to a situation in which the integrity of the arachnoid granulations is more severely affected by haemorrhagic damage. According to our recent study, a lower BMI was associated with a progressive increase in the ventricle volume during the early periods after SAH^2^. BMI and BMD are positively correlated^19,20^. In addition, previous studies have described that BMI is associated with cerebral haemorrhages that are induced by rupture of vascular smooth muscle cells that are composed of collagen type 1^21–23^.

We investigated trends in ventricle size changes after SAH with/without hydrocephalus-related symptoms, but progression of brain atrophy induced by SAH may have affected ventricular dilation. Ventricular enlargement can be present in both hydrocephalus and subcortical brain atrophy cases^24^. Previous studies have reported that global cerebral atrophy can be induced by SAH and that older age was significantly associated with brain atrophy^25,26^. Global cerebral atrophy is often classified into subcortical atrophy, reflecting ventricular dilation, and cortical atrophy, reflecting the dilation of cortical sulci^27^. Measuring cortical atrophy after SAH is difficult, because SAH is frequently accompanied by cerebral oedema and haematoma, which distributes throughout the Sylvian fissure and cortical sulci during the early stages. However, according to the above reports, in our study, the ventricle size after SAH may reflect subcortical atrophy, particularly in older patients. Since SAH induces global cerebral atrophy, subcortical atrophy after SAH may reflect global cerebral atrophy in these patients. Therefore, in this study, ventricular enlargement after SAH in younger patients may be more related to hydrocephalus and ventriculomegaly, while that in older patients may be more associated with cerebral atrophy. Our study also demonstrated that age was an independent predictor of ventricle dilatation after SAH in the older age group. Further studies are required to investigate the association between bone density and brain atrophy after SAH.

Our study also showed that a higher initial Hunt–Hess grade was strongly associated with ventriculomegaly in younger patients. Age and ventriculomegaly on admission were associated with ventricle dilatation after SAH in older patients. A recent meta-analysis also reported that old age, high Hunt–Hess grades, low GCS scores, and acute hydrocephalus at admission were predictors of shunt-dependent hydrocephalus after SAH^28^. The non-significant association between ventriculomegaly and higher Hunt–Hess grades in older patients may be explained by a higher probability of early death, particularly in the elderly. The integrity of arachnoid granulations may be weaker in older than in younger patients. Therefore, arachnoid granulations and trabeculae may be more damaged after SAH in old age, which may increase the likelihood of ventriculomegaly development. Restoration of enlarged ventricles after haematoma resolution may also be prolonged, or the enlargement may progress in elderly patients.

This study had some limitations. First, due to the retrospective nature of this study, the time between SAH and follow-up CT images was not regular. However, even in a prospective scheduled study of ventricular dilation after SAH, it would be difficult to maintain a regular time interval between SAH occurrence and follow-up imaging, because of unexpected variables during the follow-up period, including sudden neurological deterioration, emergency external ventricular CSF drainage or shunt surgery, and vasospasm. Second, measurement errors may have occurred, especially in patients with a narrow intercortical space in the frontal bone. However, all brain CT images were magnified when measuring HU values, and all patients had spongy bone spaces between the cortical bones on these images. To reduce measurement errors, we investigated HU values in four areas of the frontal skull and averaged those HU values. In addition, the BCI and Evans ratios are extensively used and are familiar to neurosurgeons. Due to the relatively small sample size in the study, we did not encounter hyperostosis frontalis interna patients with SAH; measurement of the HU values in the frontal skull in these patients may be challenging. Third, we included only Korean patients, and this may limit the generalizability of the results. However, data consistency and accuracy due to similarity in the treatment and environmental conditions may be strengths of a single centre study, because surgical techniques and SAH management protocols may vary between hospitals, with confounding effects.

In conclusion, we sought to evaluate the relationship between HU values of the skull and ventriculomegaly in patients who had undergone clipping for SAH due to aneurysmal rupture, at a single institution, during an almost 9-year period. We found that the first tertile of skull HU values was associated with an increased risk of ventricular dilation after SAH, as compared to that in younger patients. These findings may facilitate prediction of ventriculomegaly development after SAH in younger patients, based on a convenient method of measuring HU of the frontal skull on CT at admission. Nevertheless, further large-scale studies are required to confirm these results.

## Methods

### Patient selection

Data of spontaneous aneurysmal SAH patients who underwent clipping (>18 years old) from 2002 to the present were extracted from the SAH registry of our hospital, which was designed for research purposes. The data quality, consistency, and accuracy of our registry are reliable because experienced staff manages all operative data directly and consistently; the data include intraoperative microscopic videos and photographs obtained within a single hospital. Due to the preference for use of clipping in SAH patients in our hospital, we excluded the few coiling cases to reduce treatment heterogeneity.

Two-hundred and fifty-three spontaneous aneurysmal SAH patients attending our institution from January 1, 2008 to June 31, 2016, were initially identified. There were no patients with a previous history of spontaneous SAH. We excluded 21 patients from this study for the following reasons: (1) absence of follow-up CT, and (2) postoperative haemorrhagic complications. Finally, the remaining 232 consecutive cases who underwent aneurysmal clipping for primary spontaneous SAH were included in this study. In all cases, surgical clipping was performed within 24 hours after confirmation of spontaneous aneurysmal SAH via CT angiography in the emergency room.

This study was approved by the Institutional Review Board of our hospital and conformed to the tenets of the Declaration of Helsinki. Given the retrospective nature of the study, the need to obtain informed patient consent was waived.

### Surgical treatment and management for SAH

Two faculty neurosurgeons performed all aneurysmal clippings. Operation techniques and management of SAH patients were identical for both neurosurgeons: a junior neurosurgeon was trained and supervised by a senior neurosurgeon in surgical techniques and SAH management. We treated patients with the standard SAH protocol, including triple-H therapy (hypertension, hypervolemia, haemodynamic), intracranial pressure control (mannitol), and administration of nimodipine and an anticonvulsant agent. According to our departmental policy, we performed external ventricular cerebrospinal fluid (CSF) drainage or ventriculo-peritoneal (V-P) shunt procedures instead of lumbar CSF drainage in all patients presenting with hydrocephalus to avoid the potential risk of brain herniation^29^.

### Definition of the baseline and follow-up imaging

All CT images (slice thicknesses, 4.0–5.0 mm) were obtained with a CT scanner (Siemens Flash 128, München, Germany) in our hospital. The baseline CT images were obtained in the emergency room at the time of SAH diagnosis. We included one follow-up CT image per individual; we chose the one closest to 1-year post-clipping for aneurysmal SAH. For patients that underwent external ventricular CSF drainage or the V-P shunt procedure, we included the follow-up CT images, which were taken immediately prior to surgery. The duration (days) between SAH occurrence and the follow-up CT image was investigated in all patients.

### Measurement of the skull HU value, BCI, and Evans ratio

**Figure 5:**
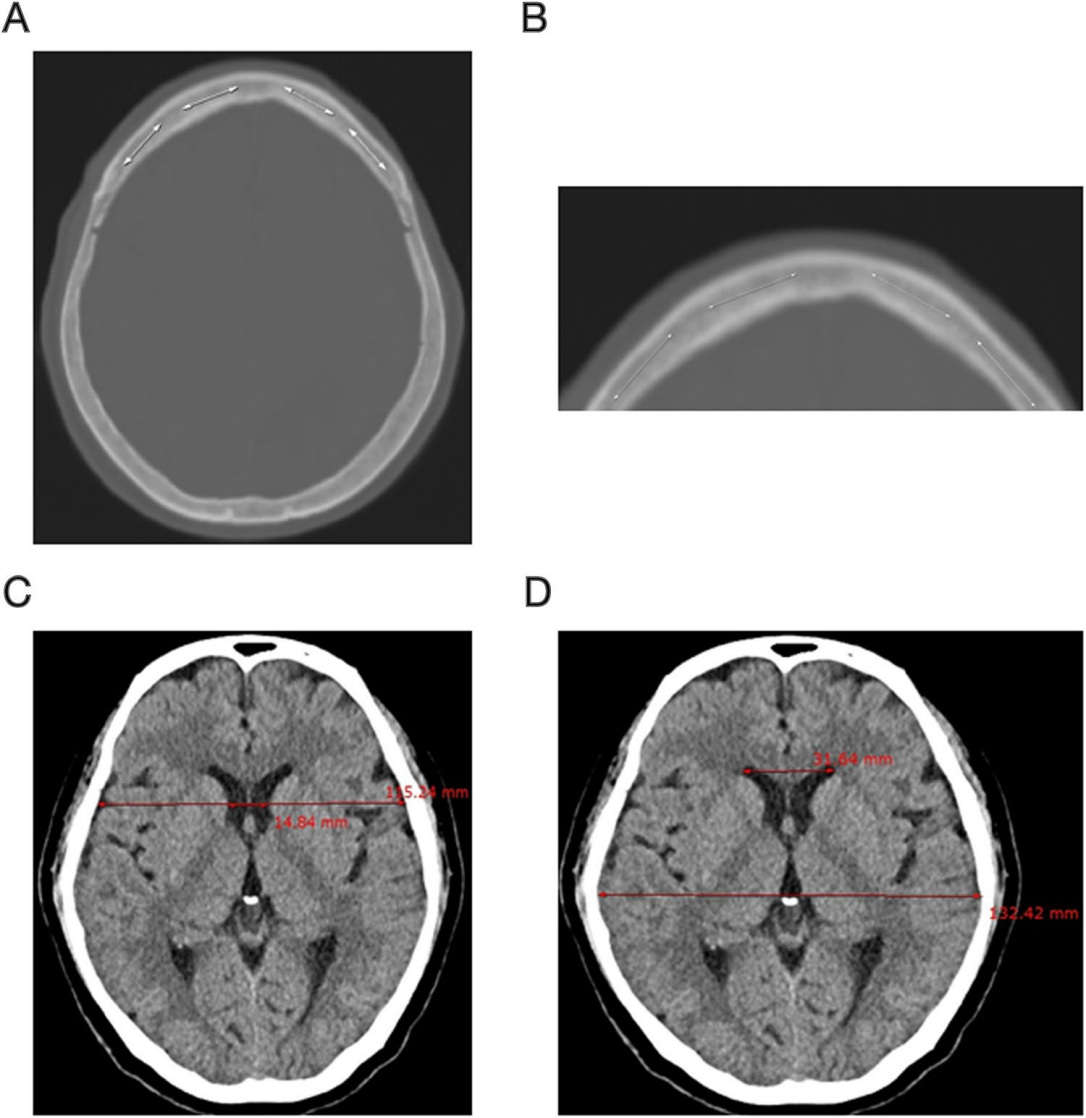
Measurement of the skull HU values, BCI, and Evans ratio: (**A**) measurement of HU values in four areas between the left and right coronal sutures of the cancellous bone of the frontal skull; (**B**) magnification of the frontal skull to measure HU values in cancellous bone, avoiding cortical bone; (**C**) measurement of the BCI by dividing the width of the frontal horns, at the level of the caudate nuclei, by the corresponding diameter of the brain at the level of the foramen Monro; (**D**) measurement of the Evans ratio with a linear ratio between the maximal width of the frontal horns in relation to the inner diameter of the cranium. HU = Hounsfield unit; BCI = bicaudate index.

All radiological evaluations were conducted by two faculty neurosurgeons blinded to the clinical data of all patients. The frontal skull HU value was measured using the baseline CT image. We measured the average HU value in the cancellous bone of the frontal skull with line drawings, using the “Linear histogram graph” function of the picture archiving and communication system (PACS) in our hospital (Fig. 5). The PACS automatically calculates and provides the maximum, minimum, and average HU values according to the line drawn. We used an axial CT slice for the measurement of the HU values in the frontal skull, immediately where the lateral ventricles disappear, or one or two slices above or below this slice. To reduce variations of HU values according to regional skull HU value heterogeneity, we attempted to set a relatively constant location for the HU value measurement. We opted for the CT slice level around where the lateral ventricles disappear, as this slice was easy to find and showed relatively thick cancellous bone in the frontal skull. All brain CT images, with a bone setting, were magnified for measurement of the HU values in the cancellous bone of the frontal skull to avoid including cortical bone, particularly in patients with a narrow intercortical space in the frontal skull. The HU values were obtained at four areas in the frontal bone to minimize measurement errors. We drew four lines along the cancellous bone of the frontal skull between the left and right coronal sutures (Fig. 5A and B). The average HU value of each of the four lines on the frontal bone was recorded in all patients (Supplementary Fig. S4).

The BCI and Evans ratio were measured using both baseline and follow-up CT images. The BCI was measured by dividing the width of the frontal horns, at the level of the caudate nuclei, by the corresponding diameter of the brain at the level of the foramen Monro. If the foramen Monro were between two CT sections, the mean value for the BCI was recorded (Fig. 5C)^30^. The Evans ratio was defined as a linear ratio between the maximal width of the frontal horns in relation to the inner diameter of the cranium (Fig. 5D)^31^.

### Clinical and radiographic variables

In addition to the information in the SAH registry, electronic medical records of all patients were reviewed. Body weight and height were recorded on admission to determine the anaesthetic dose and body mass index (BMI) was calculated as weight/(height × height) and expressed in kg/m^2^. Hunt and Hess and modified Fisher grades were included to determine the severity of SAH. We defined pan-ventricular haemorrhage as the presence of haematoma in both the lateral, and third and fourth ventricles, simultaneously. The operation type, V-P shunt, and fenestration of the lamina terminalis were identified through the records of our registry and operative records. Vasospasm was defined as having neurological symptoms with angiographic spasms on follow-up digital subtraction angiography, CT angiography, or perfusion CT.

### Statistical methods

Continuous variables were expressed as the mean ± standard deviation or median (interquartile ranges), while discrete variables were expressed as a count with percentage. The chi-square test and Student’s *t*-test were used to assess clinical differences between two groups categorized by age.

A scatterplot with a regression line or a line determined by a locally weighted scatter plot smoothing (LOWESS) was used to represent the association between several variables graphically. We used the frontal skull HU as the average value between four HU values of the frontal bone in the analysis. To determine the optimal cut-off value of age that may be associated with frontal skull HU, we used a receiver operating characteristic curve with the frontal skull HU divided by the median as the dependent variable.

CT images that met BCI > 0.2 and Evans > 0.3 simultaneously were defined as ventricular enlargement and were included in statistical analysis. The cumulative hazard for ventriculomegaly was examined using Kaplan–Meier analysis based on the skull HU value categories, with censoring of patients lacking ventricular enlargement on the follow-up CT images. Hazard ratios with 95% confidence intervals were then calculated using Cox regression analysis to identify independent factors predictive of the risk of ventricular enlargement after clipping for ruptured aneurysmal SAH. A p value < 0.05 was considered statistically significant.

All statistical analyses were performed using R version 3.3.3 (https://www.r-project.org/).

### Data availability

All data generated or analysed during this study are included in this published article (and its Supplementary Information files).

## Electronic supplementary material

Supplementary information

## References

[1] Chen S, Luo J, Reis C, Manaenko A, Zhang J (2017). Hydrocephalus after Subarachnoid Hemorrhage: Pathophysiology, Diagnosis, and Treatment. BioMed Res. Int..

[2] Bang, J. H. *et al*. Factors predicting ventricle volume increase after aneurysmal clipping in patients with subarachnoid hemorrhage. *World Neurosurg*., 10.1016/j.wneu.2017.08.076 (2017). 10.1016/j.wneu.2017.08.076 28842230

[3] Lee S, Chung CK, Oh SH, Park SB (2013). Correlation between Bone Mineral Density Measured by Dual-Energy X-Ray Absorptiometry and Hounsfield Units Measured by Diagnostic CT in Lumbar Spine. J. Korean Neurosurg. Soc..

[4] Schreiber JJ, Anderson PA, Rosas HG, Buchholz AL, Au AG (2011). Hounsfield units for assessing bone mineral density and strength: a tool for osteoporosis management. J. Bone Joint Surg. Am..

[5] Choi MK, Kim SM, Lim JK (2016). Diagnostic efficacy of Hounsfield units in spine CT for the assessment of real bone mineral density of degenerative spine: correlation study between T-scores determined by DEXA scan and Hounsfield units from CT. Acta Neurochir. (Wien).

[6] Schreiber JJ, Anderson PA, Hsu WK (2014). Use of computed tomography for assessing bone mineral density. Neurosurg. Focus.

[7] Goldman LW (2007). Principles of CT and CT Technology. J. Nucl. Med. Technol..

[8] Timothy, G. F. *The Mathematics of Medical Imaging -* A Beginner’s Guide (eds Jonathan, M. B. *et al*.) Ch. 1, 1–10 (Springer, 2010).

[9] Pickhardt PJ (2013). Opportunistic screening for osteoporosis using abdominal computed tomography scans obtained for other indications. Ann. Intern. Med..

[10] Wang, Q., Reganti, N., Yoshioka, Y., Howell, M. & Clement, G. T. Comparison between diffuse infrared and acoustic transmission over the human skull. *Proc*. *Meet*. *Acoust*. *Acoust*. *Soc*. *Am*. **22** (2015). 10.1121/2.0000005 PMC4286808 25580181

[11] Chen S (2014). Controversies and evolving new mechanisms in subarachnoid hemorrhage. Prog. Neurobiol..

[12] Glimcher SA, Holman DW, Lubow M, Grzybowski DM (2008). *Ex Vivo* Model of Cerebrospinal Fluid Outflow across Human Arachnoid Granulations. Invest. Ophthalmol. Vis. Sci..

[13] Saboori P, Sadegh A (2015). Histology and Morphology of the Brain Subarachnoid Trabeculae. Anat. Res. Int..

[14] Brandi ML (2009). Microarchitecture, the key to bone quality. Rheumatol. Oxf. Engl..

[15] Grant SF (1996). Reduced bone density and osteoporosis associated with a polymorphic Sp1 binding site in the collagen type I alpha 1 gene. Nat. Genet..

[16] Charnas LR, Marini JC (1993). Communicating hydrocephalus, basilar invagination, and other neurologic features in osteogenesis imperfecta. Neurology.

[17] Debette S (2014). Familial occurrence and heritable connective tissue disorders in cervical artery dissection. Neurology.

[18] Gaberel T (2016). Ruptured intracranial aneurysm in patients with osteogenesis imperfecta: 2 familial cases and a systematic review of the literature. Neurochirurgie..

[19] Ravn P (1999). Low body mass index is an important risk factor for low bone mass and increased bone loss in early postmenopausal women. Early Postmenopausal Intervention Cohort (EPIC) study group. J. Bone Miner. Res. Off. J. Am. Soc. Bone Miner. Res..

[20] Kim SJ, Yang W-G, Cho E, Park E-C (2012). Relationship between Weight, Body Mass Index and Bone Mineral Density of Lumbar Spine in Women. J. Bone Metab..

[21] Biffi A (2011). Body mass index and etiology of intracerebral hemorrhage. Stroke.

[22] Lioutas V-A (2017). Lacunar Infarcts and Intracerebral Hemorrhage Differences: A Nested Case-Control Analysis in the FHS (Framingham Heart Study). Stroke.

[23] Lengfeld J (2012). Protein kinase C δ regulates the release of collagen type I from vascular smooth muscle cells via regulation of Cdc42. Mol. Biol. Cell.

[24] Fishman RA, Dillon WP (2001). Normal Pressure Hydrocephalus: New Findings and Old Questions. Am. J. Neuroradiol..

[25] Tam AKH (2013). Impact of global cerebral atrophy on clinical outcome after subarachnoid hemorrhage. J. Neurosurg..

[26] Tam AKH, Ilodigwe D, Li Z, Schweizer TA, Macdonald RL (2013). Global cerebral atrophy after subarachnoid hemorrhage: a possible marker of acute brain injury and assessment of its impact on outcome. Acta Neurochir. Suppl..

[27] Palm WM (2009). Ventricular Dilation. Ann. Neurol..

[28] Xie, Z. *et al*. Predictors of Shunt-dependent Hydrocephalus After Aneurysmal Subarachnoid Hemorrhage? A Systematic Review and Meta-Analysis. *World Neurosurg*., 10.1016/j.wneu.2017.06.119 (2017). 10.1016/j.wneu.2017.06.119 28652120

[29] Bloch J, Regli L (2003). Brain stem and cerebellar dysfunction after lumbar spinal fluid drainage: case report. J. Neurol. Neurosurg. Psychiatry.

[30] Dupont S, Rabinstein AA (2013). CT evaluation of lateral ventricular dilatation after subarachnoid hemorrhage: baseline bicaudate index values [correction of balues]. Neurol. Res..

[31] Ambarki, K. *et al*. Brain ventricular size in healthy elderly: comparison between Evans index and volume measurement. *Neurosurgery* **67**, 94–99, discussion 99 (2010). 10.1227/01.NEU.0000370939.30003.D1 20559096

