# Supplementary material for: The frontal skull Hounsfield unit value can predict ventricular enlargement in patients with subarachnoid haemorrhage
Source: Sci Rep. 2018 Jul 5;8:10178. doi: 10.1038/s41598-018-28471-1 (PMC6033863; doi:10.1038/s41598-018-28471-1)
Supplement: Supplementary file 1 — Supplementary information [file 41598_2018_28471_MOESM1_ESM.docx]

**Supplementary information**

**The frontal skull Hounsfield unit value can predict ventricular enlargement in patients with subarachnoid haemorrhage**

Authors: Yu Deok Won, Min Kyun Na, Choong Hyun Kim, Jae Min Kim, Jin Hwan Cheong, Je il Ryu, Myung-Hoon Han

**Supplementary data:**

**Figures S1-~~4~~**

**Tables S1-3**


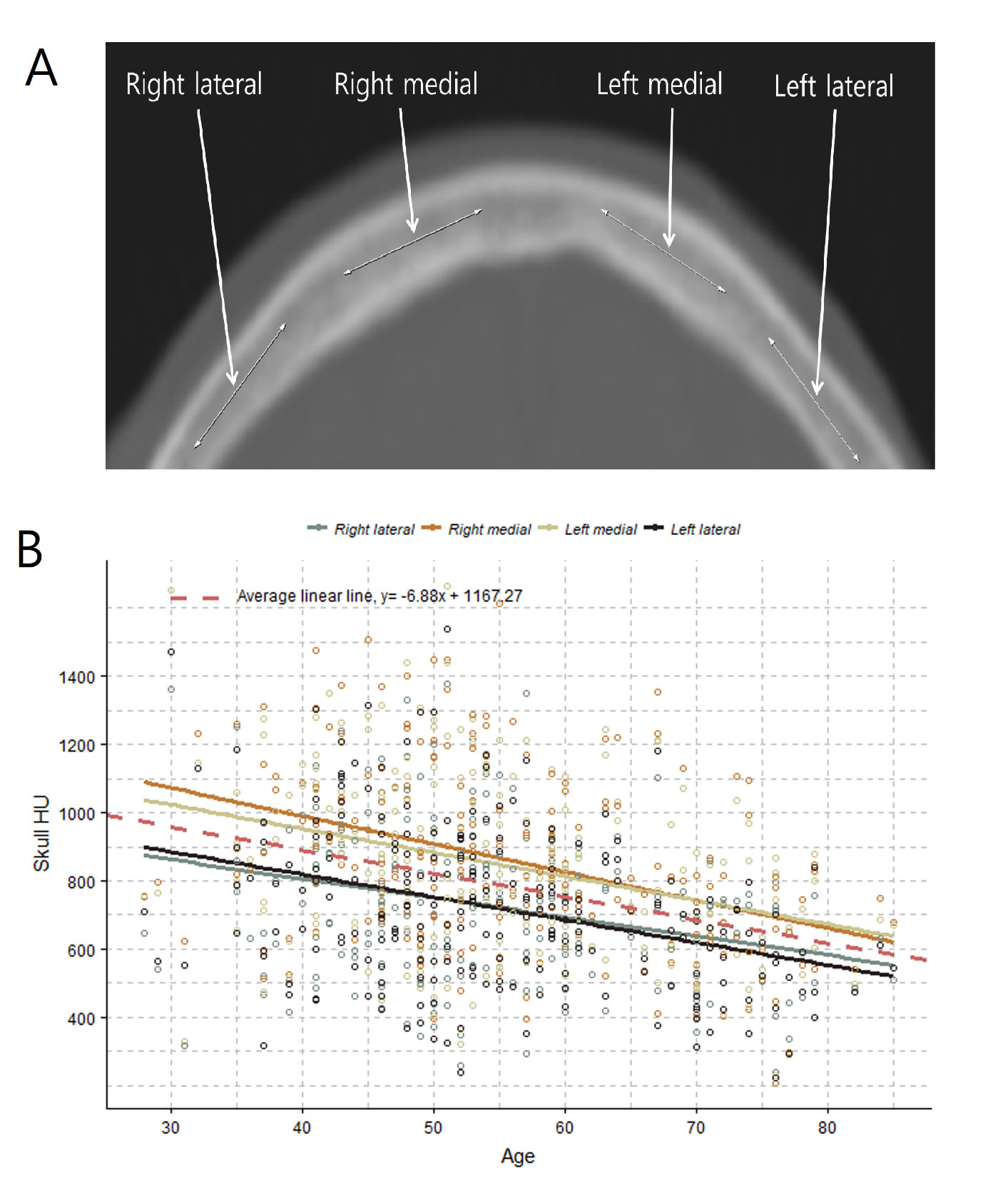


**Supplementary Fig. S1.** The association between each four HU of the frontal bone and age.


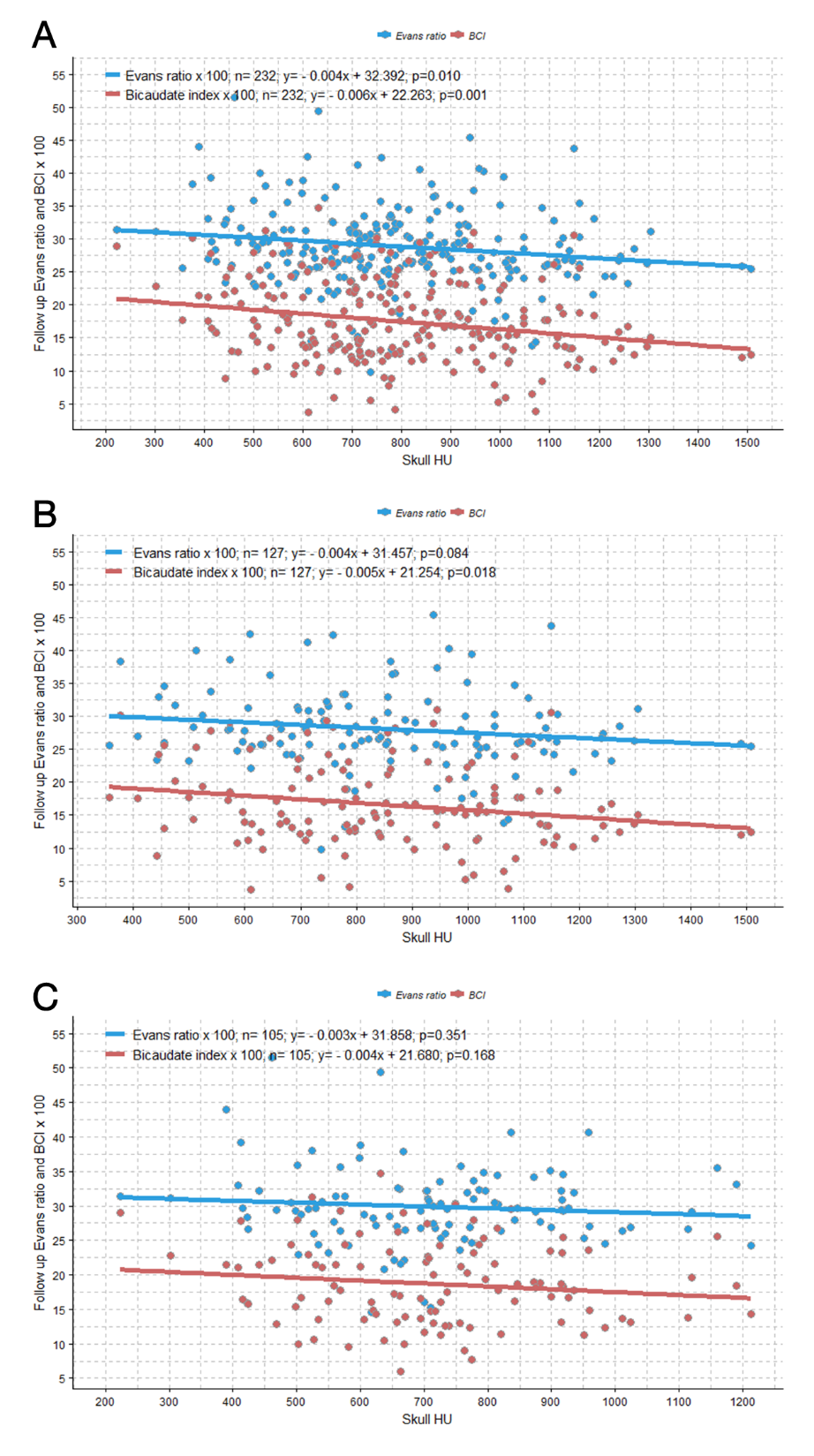


**Supplementary Fig. S2.** Scatterplot with linear regression line showing the association between the skull HU and follow up bicaudate index (x100), and Evans ratio (x100) after surgical clipping for SAH: (A) all patients; (B) younger patients; (C) older patients.


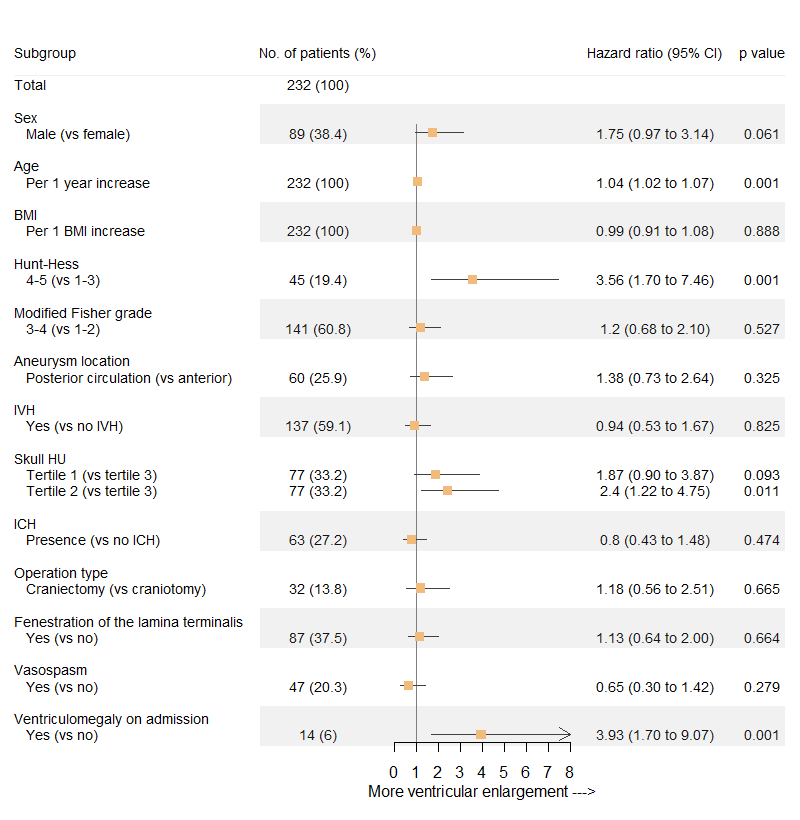


**Supplementary Fig. S3.** Forest plots of estimates from the multivariate Cox regression of ventriculomegaly according to the potential predictive factors (adjusted for sex, age [continuous variable], BMI [continuous variable], Hunt-Hess grade, modified Fisher grade, aneurysm location, IVH, skull HU, ICH, operation type, fenestration of the lamina terminalis, vasospasm, and ventriculomegaly on admission). BMI=body mass index; IVH=intraventricular hemorrhage; HU=Hounsfield unit; ICH=intracerebral hemorrhage.

**
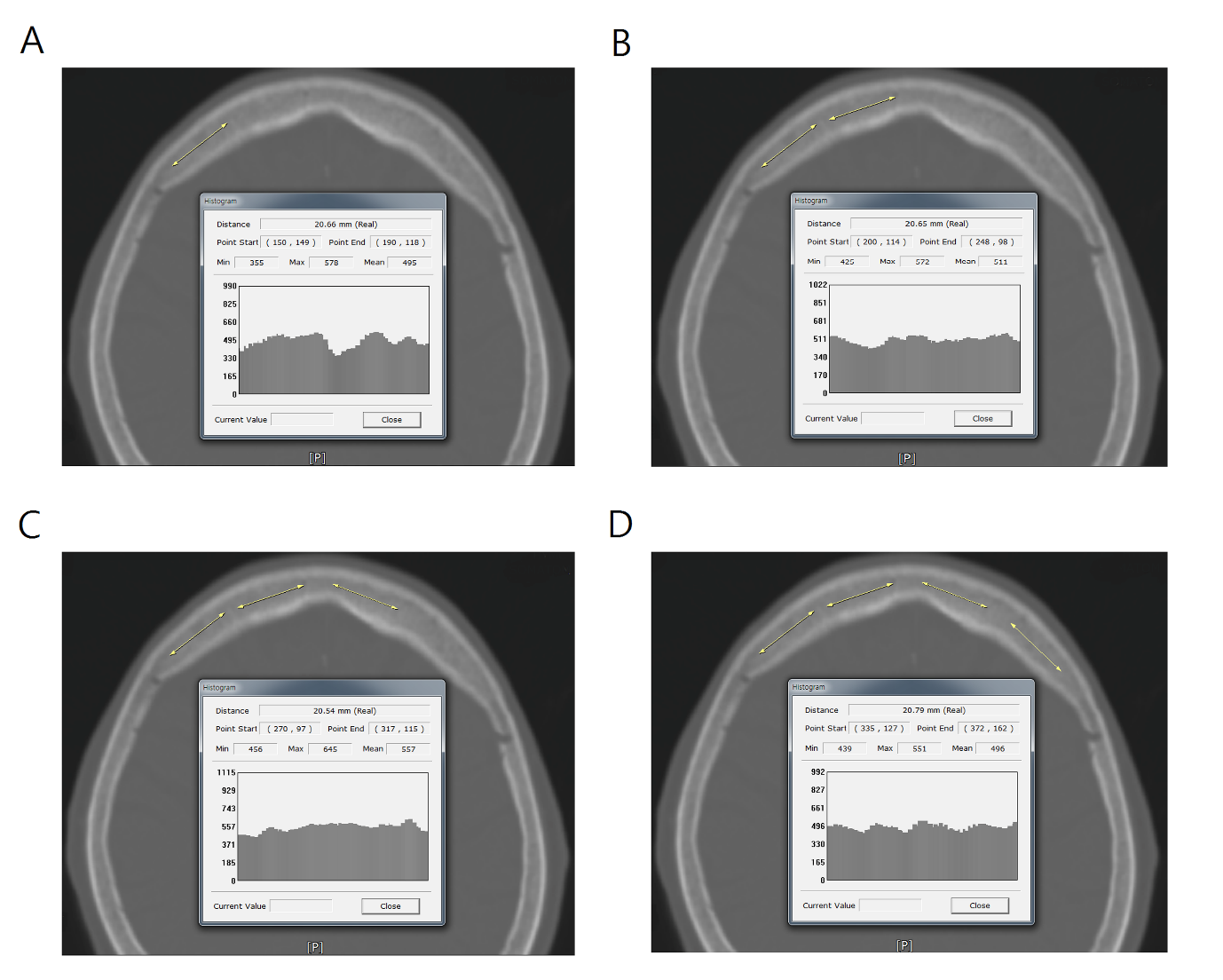
**

**Supplementary Fig. S4.** The average HU value of each of the four lines on the frontal bone. The PACS automatically calculates and provides the maximum, minimum, and average HU values according to the drawing line. HU=Hounsfield unit; PACS= picture archiving and communication system.

**Supplementary Table S1.** Univariate Cox regression analysis of ventriculomegaly after aneurysmal clipping for spontaneous subarachnoid hemorrhage for various predictive factors in all patients

|  | Univariate Cox regression analysis | | |
| --- | --- | --- | --- |
| Variable | HR | 95%CI | p |
| Sex |  |  |  |
| Male | 0.99 | 0.61–1.59 | 0.957 |
| Female |  | Reference |  |
|  |  |  |  |
| Age (per 1-year increase) | 1.04 | 1.02–1.06 | < 0.001 |
|  |  |  |  |
| BMI (per 1 BMI increase) | 0.96 | 0.89–1.03 | 0.272 |
|  |  |  |  |
| Hunt-Hess |  |  |  |
| 1–3 |  | Reference |  |
| 4 and 5 | 3.37 | 1.97–5.76 | < 0.001 |
| Modified Fisher grade |  |  |  |
| 1 and 2 |  | Reference |  |
| 3 and 4 | 1.80 | 1.08–2.99 | 0.025 |
| Aneurysm location |  |  |  |
| Anterior circulation |  | Reference |  |
| Posterior circulation | 1.32 | 0.78–2.23 | 0.308 |
| IVH |  |  |  |
| No |  | Reference |  |
| Yes | 1.61 | 0.98–2.63 | 0.060 |
| Skull HU, tertile group |  |  |  |
| Tertile 1 | 3.11 | 1.62–5,96 | 0.001 |
| Tertile 2 | 2.59 | 1.37–4.90 | 0.004 |
| Tertile 3 |  | Reference |  |
| ICH |  |  |  |
| No |  | Reference |  |
| Presence | 0.94 | 0.55–1.59 | 0.937 |
| Operation type |  |  |  |
| Craniotomy |  | Reference |  |
| Craniectomy | 2.17 | 1.23–3.85 | 0.008 |
| Fenestration of the lamina terminalis |  |  |  |
| No |  | Reference |  |
| Yes | 1.11 | 0.68–1.81 | 0.684 |
| Vasospasm |  |  |  |
| No |  | Reference |  |
| Yes | 0.51 | 0.25–1.07 | 0.074 |
| Ventriculomegaly on admission |  |  |  |
| No |  | Reference |  |
| Yes | 6.10 | 2.92–12.75 | < 0.001 |

**Supplementary Table S2.** Univariate Cox regression analysis of ventriculomegaly after aneurysmal clipping for spontaneous subarachnoid hemorrhage for various predictive factors among patients under 55 years old

|  | Univariate Cox regression analysis | | |
| --- | --- | --- | --- |
| Variable | HR | 95%CI | p |
| Sex |  |  |  |
| Male | 1.35 | 0.66–2.78 | 0.413 |
| Female |  | Reference |  |
|  |  |  |  |
| Age (per 1-year increase) | 1.07 | 1.00–1.14 | 0.041 |
|  |  |  |  |
| BMI (per 1 BMI increase) | 1.04 | 0.94–1.15 | 0.447 |
|  |  |  |  |
| Hunt-Hess |  |  |  |
| 1–3 |  | Reference |  |
| 4 and 5 | 7.44 | 3.35–16.51 | < 0.001 |
| Modified Fisher grade |  |  |  |
| 1 and 2 |  | Reference |  |
| 3 and 4 | 1.43 | 0.70–2.93 | 0.327 |
| Aneurysm location |  |  |  |
| Anterior circulation |  | Reference |  |
| Posterior circulation | 1.08 | 0.41–2.81 | 0.878 |
| IVH |  |  |  |
| No |  | Reference |  |
| Yes | 2.00 | 0.98–4.11 | 0.059 |
| Skull HU, tertile group |  |  |  |
| Tertile 1 | 3.31 | 1.17–9.41 | 0.024 |
| Tertile 2 | 2.84 | 1.02–7.95 | 0.046 |
| Tertile 3 |  | Reference |  |
| ICH |  |  |  |
| No |  | Reference |  |
| Presence | 1.00 | 0.46–2.18 | 0.992 |
| Operation type |  |  |  |
| Craniotomy |  | Reference |  |
| Craniectomy | 2.39 | 1.04–5.45 | 0.039 |
| Fenestration of the lamina terminalis |  |  |  |
| No |  | Reference |  |
| Yes | 1.08 | 0.52–2.24 | 0.828 |
| Vasospasm |  |  |  |
| No |  | Reference |  |
| Yes | 0.37 | 0.11–1.21 | 0.099 |
| Ventriculomegaly on admission |  |  |  |
| No |  | Reference |  |
| Yes | 8.76 | 2.00–38.40 | 0.004 |

**Supplementary Table S3.** Univariate Cox regression analysis of ventriculomegaly after aneurysmal clipping for spontaneous subarachnoid hemorrhage for various predictive factors among patients 55 years old or older

|  | Univariate Cox regression analysis | | |
| --- | --- | --- | --- |
| Variable | HR | 95%CI | p |
| Sex |  |  |  |
| Male | 1.19 | 0.54–2.60 | 0.667 |
| Female |  | Reference |  |
|  |  |  |  |
| Age (per 1-year increase) | 1.05 | 1.02–1.09 | 0.006 |
|  |  |  |  |
| BMI (per 1 BMI increase) | 0.90 | 0.81–0.99 | 0.029 |
|  |  |  |  |
| Hunt-Hess |  |  |  |
| 1–3 |  | Reference |  |
| 4 and 5 | 1.58 | 0.72–3.47 | 0.253 |
| Modified Fisher grade |  |  |  |
| 1 and 2 |  | Reference |  |
| 3 and 4 | 2.06 | 0.97–4.36 | 0.059 |
| Aneurysm location |  |  |  |
| Anterior circulation |  | Reference |  |
| Posterior circulation | 1.17 | 0.61–2.26 | 0.636 |
| IVH |  |  |  |
| No |  | Reference |  |
| Yes | 1.00 | 0.51–1.98 | 0.997 |
| Skull HU, tertile group |  |  |  |
| Tertile 1 | 1.77 | 0.82–3.79 | 0.144 |
| Tertile 2 | 1.31 | 0.58–2.97 | 0.523 |
| Tertile 3 |  | Reference |  |
| ICH |  |  |  |
| No |  | Reference |  |
| Presence | 0.85 | 0.41–1.74 | 0.65 |
| Operation type |  |  |  |
| Craniotomy |  | Reference |  |
| Craniectomy | 2.09 | 0.94–4.64 | 0.071 |
| Fenestration of the lamina terminalis |  |  |  |
| No |  | Reference |  |
| Yes | 1.32 | 0.66–2.63 | 0.436 |
| Vasospasm |  |  |  |
| No |  | Reference |  |
| Yes | 0.69 | 0.27–1.77 | 0.439 |
| Ventriculomegaly on admission |  |  |  |
| No |  | Reference |  |
| Yes | 4.24 | 1.77–10.18 | 0.001 |
